# American Dental Association Meeting

**Published:** 1895-09

**Authors:** 


### EDITORIAL.

### American Dental Association Meeting.

The thirty-fifth annual meeting of this body was held in Asbury Park, August 6th to the 9th inclusive. About two hundred members were present and quite a large number of visitors. The meeting was, all things considered, a good one; a number of excellent papers were read and discussed—instructive and entertaining. The spirit of the meeting was good, notwithstanding some unfavorable conditions. Asbury Park is not a desirable place to hold such a meeting. Suitable accommodations were not afforded the members and visitors; no headquarters were provided, and indeed it was said could not be, on account of the great crowds of people present; and the dentists were distributed, a few here, a few there, all over the place; some were compelled to occupy quite undesirable places so that the social feature of the occasion was almost wholly lost. Another discouragement was found in the place of meeting—the Auditorium, a large glass building, four times too large for the meeting, which was held in the center, and a wall of exhibits completely surrounding it, so that it was simply impossible to secure proper quiet and order. The exhibitors were not responsible for this, so much as persons who were not interested in the meeting, were on the outskirts, and were necessarily the occasion of more or less stir and confusion.

A very sad feature of the occasion was the severe illness of the President, Dr. J. Y. Crawford, who was present at only the opening session and was even then very ill. He went from the hall to his bed, and was not out of his room again during the meetings. This brought a feeling of sadness over all present. The sympathy that was in the heart of every one, was shown in the fact that Dr. Crawford was, by an almost unanimous vote, elected President of the Association for next year.

The officers elected for the ensuing year are as follows: President, Dr. J. Y. Crawford, of Nashville, Tenn.; First Vice-President, Dr. James McManus, of Hartford, Conn.; Second Vice-President, Thomas Fillebrown, of Boston, Mass.; Secretary, Dr. Geo. H. Cushing, of Chicago, Ill.; Corresponding Secretary, Dr. Emma Eames Chase, of St. Louis, Mo.; Treasurer, Dr. H. W. Morgan, of Nashville, Tenn.; Members of the Executive Committee for three years: Dr. J. N. Crouse, of Chicago, Dr. L. Ottofy, of Chicago, and Dr. V. H. Jackson, of New York.

Saratoga was elected as the next place of meeting, first Tuesday of August, 1896.

There was quite a feeling against the Association being taken to the far East, the third time in succession; it certainly looks so to one from the West. Saratoga is not the best place in the country for either a scientific or professional meeting; there are too many outside attractions (fuss and feathers) to attain the best results in scientific work. But perhaps it will come out all right, and to attain that end, we suggest that every member consider himself a committee of one to work for the largest and best meeting of this body ever held. So may it be.

